# Mapping the conceptualisation, contributing factors and interventions of moral distress among healthcare providers in Africa: a scoping review

**DOI:** 10.1136/bmjgh-2025-023358

**Published:** 2026-08-04

**Authors:** Joyline Jepkosgei, Dorothy Oluoch, Jacinta Nzinga, Kate McNeil, Yingxi Zhao, Sassy Molyneux

**Affiliations:** 1 Health Services Unit, KEMRI-Wellcome Trust Research Programme Nairobi, Nairobi, Kenya; 2 Centre for Global Health Research, Nuffield Department of Medicine, University of Oxford, Oxford, UK; 3 Liverpool School of Tropical Medicine, Liverpool, UK; 4 Pandemic Sciences Institute, University of Oxford, Oxford, UK; 5 KEMRI-Wellcome Trust Research Programme, Kilifi, Kenya

**Keywords:** Africa, Health systems, Health Personnel, Review

## Abstract

**Introduction:**

Moral distress is the psychological discomfort experienced by healthcare providers when prevented from acting on their ethical values. Although extensively studied in high-income settings, it remains relatively unexplored in Africa, where chronic resource limitations and systemic constraints shape clinical practice. This scoping review mapped African literature examining how moral distress is conceptualised and discussed, its drivers and interventions implemented to address it with a particular focus on the balance between individual and organisational perspectives.

**Methods:**

A systematic search of databases identified papers on moral distress and related concepts among healthcare providers in Africa. Data extracted included: publication year, geographical focus, study design, participants, healthcare setting, conceptual framing, definitions, factors contributing to moral distress and descriptions of interventions and their evaluations. The results were analysed thematically considering individual and organisational perspectives.

**Results:**

30 papers published between 1999 and 2025 across 11 African countries were included. Of these, 14 were qualitative, 10 quantitative, 2 mixed methods, 1 review, 2 commentaries and 1 book chapter. Moral distress was commonly framed as an individual psychological burden, yet its primary drivers were identified as organisational including staff shortages, excessive workloads, inadequate resources and limited institutional support. Quantitative papers explicitly referred to ‘moral distress’, while qualitative papers generally described ethical and moral challenges more widely. Reported interventions were mainly proposed: counselling, debriefing, ethics committees and better staffing were recommended but rarely implemented or evaluated. In practice, healthcare workers relied on informal coping strategies such as peer support, prayer and improvisation.

**Conclusion:**

Moral distress is increasingly explored in the literature on African health systems but remains inconsistently defined and inadequately addressed at the organisational level. Despite widespread acknowledgement of its organisational and systemic origins, responsibility for managing its effects falls largely on individuals. This review supports a systems-level understanding of moral distress and highlights the need for organisational and policy change processes. Future research should move beyond description to identifying and evaluating team-based and organisational strategies that effectively reduce or manage moral distress.

**WHAT IS ALREADY KNOWN ON THIS TOPIC**

- Moral distress has been widely studied in high-income countries’ health systems, where it is increasingly recognised as an organisational and systemic issue linked to burnout, turnover and compromised care quality.
- In African contexts, evidence is relatively limited and has not previously been synthesised.

**WHAT THIS STUDY ADDS**

- This is the first scoping review to synthesise papers on moral distress in African health systems.
- Although organisational and systemic drivers of moral distress are acknowledged, the concept remains inconsistently defined and is often framed as an individual than as a system-level issue.
- Organisational and policy responses are rarely institutionalised, leading to heavy dependence on personal and informal relational coping strategies.

**HOW THIS STUDY MIGHT AFFECT RESEARCH, PRACTICE OR POLICY**

- Highlights the need to recognise moral distress as a signal of systemic issues and to incorporate efforts to address it into workforce resilience strategies.
- Emphasises opportunities for practice and policy to develop team, organisational and system supports to reduce reliance on individual coping strategies.
- Identifies the need for future research in Africa focused on how organisational and system conditions shape moral distress and on context-appropriate change processes.

## Introduction

 Globally, moral distress has become an increasing concern among frontline healthcare providers. Its origins date back to the 1970s and 1980s in nursing ethics. The concept emerged when nursing students, feeling overlooked by more theoretical medical ethics, voiced concerns about the practical and emotional challenges of nursing care.^1^ The term moral distress was first introduced to describe the psychological distress that arises when an individual knows the right course of action but is unable to act due to institutional constraints.^2^ This earlier conceptualisation emphasised the conflict between an individual’s values and external constraints such as institutional policies, resource limitations and societal pressure.^2 3^ Subsequent work expanded the concept to include related phenomena such as moral residue (the lingering impact of distress) and the crescendo effect, which captures the cumulative impact of repeated exposure to morally distressing situations.^4^ There is currently no consensus on a single definition of moral distress, with ongoing debates focused on the assumptions underlying the concept and its scope. Some critique the notion that a nurse’s moral judgements are always correct and that nurses are powerless to act, arguing that this reinforces a particular notion of victimhood and calling for the abandonment of this concept.^5^ Others argue that constraint-based definitions are too narrow, as they fail to capture the full range of moral experiences, including uncertainty, conflict and dilemmas.^6–8^ Despite these arguments, moral distress has been recognised as a useful concept to understand ethical challenges in healthcare settings and to promote ethically supportive environments.^9^

Empirical research on moral distress among healthcare providers has primarily been conducted in high-income settings, where early studies focused on nurses have been expanded to include physicians, social workers and other professions.^10–12^ These studies show that moral distress has significant effects across multiple levels. At the individual level, it undermines provider well-being, contributing to burnout, job dissatisfaction, absenteeism and compassion fatigue,^13 14^ with direct implications for the quality of patient care and potentially compromising compassion in clinical encounters.^15^ At the organisational level, moral distress has been shown to contribute to staff turnover and workforce attrition,^16–18^ leading to staff shortages, loss of expertise and increased recruitment and reorganisation costs.^19^ Collectively, these effects weaken organisational resilience and the overall capacity of health systems to deliver consistent care.

Reviews drawing primarily on high-income setting studies have synthesised evidence on the prevalence, severity and conceptual foundations of moral distress,^14 15^ as well as interventions developed to mitigate it, such as facilitated discussions, reflective practice, multidisciplinary ethics rounds and education interventions.^20 21^ However, in many African health systems, healthcare providers work within chronically under-resourced and structurally fragile systems, sometimes worsened by professional hierarchies and ethical dilemmas associated with poverty, inequality and limited access to care.^22–24^ These contextual factors may heighten moral distress, emphasising the need to explore interventions and change processes appropriate to the African context.

There has been little synthesis of empirical evidence in African contexts, limiting managers’ and policymakers’ ability to design context-specific interventions that support provider well-being and sustain the health workforce. Also, it remains unclear how these conceptual disputes over moral distress have been addressed in African papers. Given these gaps, we conducted a scoping review to map how moral distress has been conceptualised and discussed in African healthcare settings, with particular interest in the balance between individual and organisational perspectives. Specifically, our review explores how moral distress is conceptualised, the key drivers and the interventions identified and implemented to address moral distress.

### Research questions

The following research questions guided the review.

1. What literature exists on moral distress in Africa, and how is moral distress conceptualised in these papers?
2. What are the key drivers or contributors to moral distress among frontline healthcare providers in Africa?
3. What interventions have been implemented to address moral distress among frontline healthcare providers in Africa?

## Methods

This review was conducted in accordance with the Joanna Briggs Institute (JBI) methodology for scoping reviews^25^ and was prospectively registered on the Open Science Framework (https://osf.io/vr3uq). To enhance transparency and completeness, reporting adheres to the Preferred Reporting Items for Systematic Review and Meta-Analysis extension for Scoping Reviews (PRISMA-ScR) checklist.

### Eligibility criteria

The eligibility criteria were guided by the JBI Population, Concept, Context (PCC) framework. Papers were included if they examined moral distress and related constructs, such as moral injury and constraint among healthcare providers in African healthcare settings. Detailed inclusion and exclusion criteria are presented in online supplemental appendix table A1.

### Types of sources

This review considered empirical papers that employed qualitative, quantitative or mixed methods designs. Systematic reviews meeting the inclusion criteria were also included. Additionally, relevant editorials, commentaries and opinion papers were included if they provided conceptual or contextual insights into moral distress.

### Evidence sources

Comprehensive literature searches were conducted in the following databases: MEDLINE (Ovid), PubMed, CINAHL, EMBASE, Scopus and Web of Science. Both PubMed and Ovid MEDLINE were searched to ensure the results were complete and up to date. Additional searches were conducted in Google Scholar and by screening the reference lists of included papers to identify relevant grey literature (non-peer-reviewed papers), including dissertations, commentaries, editorials and opinion papers. These supplementary searches were undertaken as part of the literature review, and all records were screened using the same eligibility criteria as those used for the database searches.

### Search strategy

The search strategy was developed by JJ, discussed with all authors and refined in consultation with a research librarian (AM). This followed a three-step process: (1) An initial limited search of MEDLINE and CINAHL to identify relevant keywords and index terms. (2) Development of a comprehensive search strategy using identified terms and Boolean operators, which was then adapted to each database. (3) Screening of reference lists from all included articles and related reviews to identify additional records.

Synonyms and truncation were used where appropriate. Papers published in English only were included. No date limits were applied due to the limited literature available. The search was conducted across the databases on 14 March 2025. The search strategy is provided in online supplemental appendix table A1.

### Data screening, charting and synthesis

All retrieved records were imported into EndNote for deduplication (some overlaps were expected, eg, between PubMed and Ovid MEDLINE records) and subsequently uploaded to Covidence,^26^ an online review management platform for screening. JJ independently screened all titles and abstracts. A second reviewer (KM) independently reviewed a random 10% sample of the articles excluded by the first reviewer. No additional papers meeting the inclusion criteria were identified during this second review. The remaining articles were then assessed, and papers that did not meet the inclusion criteria were excluded. Data extraction was primarily conducted by JJ in NVivo 12, and the data were later charted in Excel. To enhance analytical rigour and reliability, emerging findings and patterns were discussed with all authors throughout data extraction and synthesis. The full results of the search and study selection process are presented in the findings section.

A thematic approach combining deductive and inductive theme development was employed. Findings are presented narratively and summarised in tables of study characteristics and themes.

## Results

### Literature search results

**Figure 1:**
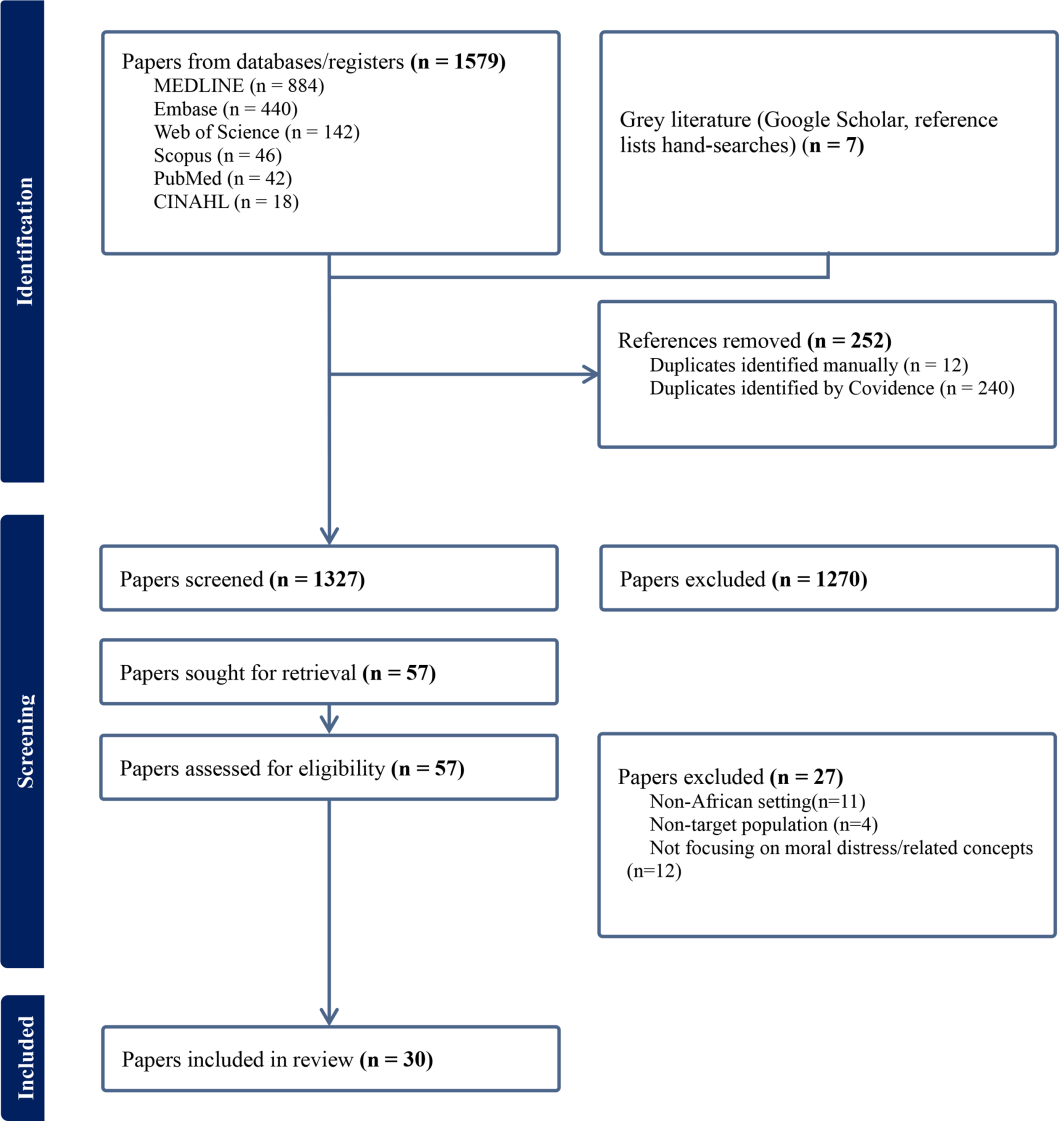
PRISMA ScR flow chart summary. PRISMA ScR, Preferred Reporting Items for Systematic Review and Meta-Analysis extension for Scoping Reviews.

The search yielded 1579 records from databases (Embase=440, MEDLINE=884, PubMed=42, CINAHL=18, Web of Science=142, Scopus=46) and 7 from grey literature. After removing 252 duplicates, 1327 records remained. During title and abstract screening, 1270 articles were excluded for failing to meet one or more predefined eligibility criteria, leaving 57 articles for eligibility assessment. Of these, 27 were excluded (11 from non-African settings, 4 targeting non-relevant populations and 12 not focusing on moral distress or related concepts). Ultimately, 30 papers were included. The process is summarised in the PRISMA-ScR flow diagram (figure 1).

### Study characteristics

We provide a summary of the characteristics of the included papers in table 1, detailing their geographical distribution, study designs and participant groups.

**Table 1:** Study characteristics summary

No.	Author(s)	Title	Country	Design/methods	Participants
1.	Beyaffers *et al*^27^	Predictors of moral distress among nurses	Ethiopia	Quantitative	Nurses (n=248)
2.	Abdeen and Attia^48^	Ethical work climate, moral courage and moral distress	Egypt	Quantitative	Nurses (n=384)
3.	Ohene *et al*^28^	Moral distress and injury during COVID-19	Ghana	Quantitative	Radiographers (n=100)
4.	Emmamally and Chiyangwa^49^	Exploring moral distress among critical care nurses	South Africa	Quantitative	ICU nurses (n=74)
5.	Chukwuorji *et al*^29^	How moral distress contributes to depression	Nigeria	Quantitative	Nurses (n=398)
6.	Ashuntantang *et al*^44^	Bedside rationing and moral distress in nephrologists	15 SSA countries	Quantitative	Nephrologists (n=40)
7.	Mwenya *et al*^30^	Determinants of moral distress among oncology providers	Kenya	Quantitative	Oncology staff (n=145)
8.	Antwi and Galanza^31^	Moral caring competency and moral distress	Ghana	Quantitative	Nurses (n=231)
9.	Getahun *et al*^32^	Moral distress and associated factors among nurses	Ethiopia	Quantitative	Nurses (n=212)
10.	Berhie *et al*^33^	Moral distress and its associated factors	Ethiopia	Quantitative	Nurses (n=412)
11.	Sibiya and Rispel^34^	Ethical dilemmas and moral distress during COVID-19	South Africa	Qualitative	ICU nurses (n=21)
12.	Voget^35^	Professional nurses’ lived experiences of moral distress	South Africa	Qualitative	Nurses (n=7)
13.	Afoko *et al*^36^	Moral distress in neonatal and paediatric care	Ghana	Qualitative	Nurses and managers (n=54)
14.	Jaffre and Lange^50^	Being a midwife in West Africa	Benin and Burkina Faso	Qualitative	Midwives and families (n=36)
15.	Boakye *et al*^51^	Moral habitability of resource-constrained obstetric settings	Ghana	Qualitative	Nurses & midwives (n=30)
16.	Solum *et al*^52^	Ethical problems in practice as experienced by Malawian student nurses	Malawi	Qualitative	Student nurses (n=10)
17.	Nagdee and de Andrade^53^	Moral injury in South African SLT&As	South Africa	Qualitative	SLT and audiologists (n=25)
18.	Maluwa *et al*^37^	Moral distress in nursing practice in Malawi	Malawi	Qualitative	Nurses (n=20)
19.	Ntseke *et al*^54^	Moral distress among critical care nurses executing DNR orders	South Africa	Qualitative	Critical care nurses (n=14)
20.	Addo *et al*^38^	Moral distress among midwives in labour wards	Ghana	Qualitative	Midwives (n=8)
21.	Harrowing and Mill^39^	Moral distress among Ugandan nurses providing HIV care	Uganda	Qualitative	Nurses (n=24)
22.	DeBoer *et al*^40^	Moral distress and resilience in oncology care	Rwanda	Qualitative	Oncology clinicians (n=22)
23.	Langley *et al*^45^	Moral distress experienced by intensive care nurses	South Africa	Qualitative	ICU nurses (n=65)
24.	Boakye^55^	No other alternative than to compromise	Ghana	Qualitative	Nurses and midwives (n=30)
25.	Afoko *et al*^46^	Moral distress in nurses in developing economies	Multiple	Integrative review	16 papers
26.	Budden^41^	Audiologists’ perceptions of ethical climate and level of moral distress	South Africa	Mixed methods	Audiologists (n=84 survey; 17 interviews)
27.	Dewey^42^	Providing care with few resources	Uganda	Mixed methods	Nurses and students (n=208; 13)
28.	Ulrich^43^	Ebola is causing moral distress among African healthcare workers	–	Commentary	–
29.	Kiddell-Monroe *et al*^47^	Humanitarian ethics in MSF	West Africa, South Sudan, Nigeria	Book chapter draws on case studies: Ebola response & humanitarian settings	–
30.	Good *et al*^56^	Clinical realities and moral dilemmas: contrasting perspectives in Kenya, Tanzania and America	Kenya, Tanzania and America	Commentary	_

DNR, do-not-resuscitate; ICU, intensive care unit; MSF, Médecins Sans Frontières; SLT&As, speech–language therapists and audiologists ; SSA, sub-Saharan Africa.

The 30 included papers, published between 1999 and 2025, varied widely in design, scope and context. Nearly half of the papers were qualitative (n=14), typically employing ethnographic approaches, in-depth interviews or focus group discussions to explore healthcare workers’ experiences and perceptions of moral distress. 10 papers employed quantitative designs, most of which used structured survey questionnaires, including the Moral Distress Scale-Revised, the Moral Distress Appraisal Scale and the Ethical Climate Questionnaire, to assess prevalence and associated factors. Two papers employed a mixed-methods design, combining surveys with qualitative interviews or open-ended responses. One integrative review synthesised evidence from multiple regions, and two were commentaries and one book chapter. No longitudinal or interventional papers were identified during the review period.

Geographically, the papers covered 11 African countries, including South Africa (n=7), Ghana (n=6), Ethiopia (n=3), Uganda (n=2), Malawi (n=2), Nigeria (n=1), Egypt (n=1), Kenya (n=1), Rwanda (n=1) and Benin/Burkina Faso (n=1). Three multi-country or regionally comparative papers incorporated African data alongside data from other regions, whereas two commentaries did not specify a country focus.

Most papers were hospital-based (n=21), particularly in critical care (n=5), maternity and obstetric units (n=4), and oncology or nephrology services (n=3). One study included participants working across multiple hospital departments (n=1), while others were conducted in HIV care settings (n=1), speech and audiology services (n=2), rural healthcare settings (n=1) and nursing training institutions (n=1). Two commentaries and one book chapter discussed the experiences of moral distress in humanitarian, pandemic and outbreak contexts (n=3).

The professional focus was predominantly on nurses and midwives (n=20), including generalists, students and those in critical care (n=5) and maternity services (n=5). Other healthcare professionals were considerably less represented (n=6); notably, nephrologists, oncologists and radiologists were less frequently represented, while allied health professionals were included in two papers. Only one study included multiple professional cadres, including nurses, registrars, clinical officers and radiologists (n=1), while two commentaries and one book chapter discussed moral distress among healthcare workers more broadly without focusing on a specific professional group (n=3).

### Emerging themes

#### Conceptualisation of moral distress

Among the 30 included papers, the majority (n=23) explicitly defined or operationalised moral distress; one study provided contextual accounts of what moral distress is perceived to be; and six papers discussed related moral constructs, such as moral injury, agency and dilemmas, without providing a formal moral distress definition. Overall, the conceptualisation of moral distress was varied, though three main dominant framings emerged. Online supplemental appendix table A2 provides a detailed summary of definitions of moral distress.

Constraint-based framing was applied in 17 papers.^27–43^ These accounts drew on Jameton’s (1984) definition of moral distress as knowing the right course of action but being unable to act due to institutional or external barriers. This framing was primarily used in survey-based papers, operationalised using moral distress scales.

an emotional phenomenon that occurs when one knows the right thing to do for the patient, but institutional constraints make it impossible to pursue that action… Jameton,^3^ cited in study by Ohene-Botwe *et al*.^28^

A smaller group of papers (n=4) adopted broader framings that extend beyond constraint to include moral conflict, uncertainty and Morley’s three-way model that emphasises the causal link between the experiences of a moral event and the psychological distress that arises^44–47^

a specific psychological response to morally challenging situations such as moral constraint or moral conflict or both.^44^

Two papers emphasised emotional or relational dimensions of moral distress in their operationalisation of the term,^48 49^ while participants in some studies described experiences of guilt, powerlessness, anger, anguish or ‘suffering’ regardless of the framing.^39^

…feelings of guilt, powerlessness and anger experienced by healthcare professionals in situations where they are unable to practice according to their professional and personal values.^49^

Suffering was the word we used… when you couldn’t give care you knew they deserved.^39^

Lastly, a smaller number of papers did not provide explicit definitions but engaged with related constructs (n=7)**,** most notably moral injury, moral agency, moral habitability and ethical problems.^50–56^ For example, speech and language therapists in South Africa described lasting psycho-emotional harms consistent with moral injury. See online supplemental appendix table A3 for definitions of all constructs related to moral distress.

### Key drivers of moral distress

In our synthesis, the drivers of moral distress and the associated interventions identified across the included papers were categorised into four overarching levels: individual, relational, organisational and system. Individual-level factors relate to healthcare providers’ emotional and psychological experiences and responses to moral distress.^57^ Relational-level factors concern the interpersonal relationships and interactions within healthcare teams and with patients and their families that shape healthcare practice, including communication, hierarchy and power dynamics.^58^ These relational influences closely intertwined with organisational drivers across the included studies. Organisational-level factors relate to processes within healthcare facilities such as staffing practices, resource availability, workplace support and managerial processes.^59^ System-level factors include broader structural, socio-economic and policy conditions that shape healthcare delivery beyond the scope of individual organisations.^60 61^ Online supplemental appendix table A4 summarises the drivers at various levels.

At the individual level, moral distress was described in eight papers. Study participants reported feelings of guilt, powerlessness and threats to their professional identity when unable to deliver care. Constraints to their actions arose from the fear of infection and inadequate protection during the Ebola and COVID-19 outbreaks,^28 43^ and the impact of insufficient training, professional inexperience and lack of confidence, which left them unprepared to meet professional expectations.^28 37 45 49^ Additionally, demographic factors were linked to moral distress: women, younger staff and those working longer hours experienced higher levels of distress.^27 28 33 54^

I felt I did not do anything … I felt very bad … I had a guilty conscience.^37^

Organisational and relational drivers of moral distress were most consistently reported in 12 papers. Organisational drivers included workforce and resource constraints, such as shortages of staff, drugs and equipment, heavy workload, unsupportive leadership and weak referral systems, identified in 10 papers.^28 37 38 40 42 44–46 49 51 55^ Relational drivers arising from end-of-life care decision-making, ambiguous or conflicting clinical directives, and perceived treatment futility were reported in four papers.^40 45 46 54^ Poor interprofessional communication and hierarchical workplace cultures that excluded nurses and midwives from decision-making were highlighted in five papers.^37 38 45 49 51^

You come on duty and you have 24 clients … you are only three midwives … what would you do?^55^

Sometimes the DNR order is verbalised but not written … if I don’t do it, the doctor will shout at us.^54^

At the system level, drivers were reported in nine papers. Poverty and patients’ inability to pay for treatment or transport were documented in five papers.^37 40 42 44 46^ Family authority and cultural or religious norms constraining timely consent or sustaining futile care were reported in four papers.^38 43 51 55^ Broader governance issues, such as corruption, political interference and denial of humanitarian access, were highlighted in three papers.^40 44 47^

Let me call my husband, my father-in-law, to consult them first, or they have to see a fetish priest before they agree [to consent for treatment]. You feel so frustrated because the woman would not agree until the husband says yes. Wait for my husband to come. (Paya)^51^

### Interventions to minimise and manage moral distress

Across the reviewed papers, 11 discussed interventions or support processes aimed at minimising and mitigating moral distress. We broadly grouped them into three categories: individual-level emotional and psychological supports; organisational-level ethical support and managerial mechanisms; and system-level structural reforms. Overall, while individual-level supports, such as peer support, spirituality and personal resilience, were described as coping mechanisms, most organisational and systems-level support strategies, such as ethics deliberation/debriefing forums and increased staffing levels, respectively, were largely unimplemented in these contexts. Online supplemental appendix table A5 summarises these interventions and support processes.

At the individual level, nine papers reported on interventions focused on strengthening emotional and psychological supports such as counselling, stress management programmes and structured debriefing, particularly in intensive and critical care settings.^28 37 45 49 54^ However, such supports were generally absent or inconsistently available, with staff instead relying on informal coping strategies such as peer discussions, collegial or family support, personal resilience, prayer and other faith-based coping strategies.^39 42 44 51^

… things that we get exposed to are deep, but there is nothing. You survive, then you become desensitised. You admit another patient, carry on, no debriefing, none expected. Someone should say “How do you feel? In future how should we deal with this?” (ICU nurse C15).^45^

Organisational and relational interventions were recommended in six papers. These included creating formal spaces for ethical reflection and guidance, such as ethics committees and unit-level forums for ethical deliberations, regular ethics education or professional development opportunities and strengthening supervision.^37 45 51 54^ Other organisational priorities recommended included ensuring equitable training and promotion practices and improving departmental resourcing.^28 37 40^

The study…demonstrated the need for unit-based ethical discussion platforms and debriefing sessions for supporting critical care nurses involved in executing DNR orders.^54^

Lastly, at the system level, nine papers underscored the need for broader structural reforms such as equitable resource allocation and fair staffing (both often constrained by system-level financing and workforce policies) and stronger institutional and professional body advocacy.^28 37 51 55^ Health workers described coping through improvisation, rationing or reframing scarcity as stewardship,^40 42 46^ yet consistently called for more sustainable reforms to address the root causes of moral distress, including strengthened leadership, governance, policy oversight and health system financing settings.^44 51^

Nurses expected that the professional bodies understood what nurses went through and should offer support to improve their welfare and rights… as narrated by participant #009: they should sometimes visit us so that they appreciate how we work with limited resources…^37^

## Discussion

This review examined how moral distress is conceptualised and discussed in papers covering African healthcare contexts. The included literature was largely centred on frontline healthcare providers, predominantly nurses, across various healthcare settings. We identified key contributing factors and the interventions implemented or recommended to address moral distress. Three main patterns emerged: variations in conceptual framing, multilevel contributing factors and healthcare providers relying on informal, individual-level coping strategies. The latter was evident despite the predominance of organisational and system-level interventions being recommended. Overall, these patterns highlight a gap between the conceptualisation of moral distress, its organisational and systemic drivers and the organisational (formal) responses that remain unimplemented. As a result, individuals are responsible for managing moral distress.

### Conceptualisation of moral distress

The conceptualisation of moral distress in the included papers ranged from a narrow, constraint-based perspective to broader models that incorporate multiple moral dimensions. While most studies drew on Jameton’s original definition, others reflected expanded frameworks, including Morley *et al*’s framework, which emphasises a causal link between moral events and psychological responses.^28 37 51 54^ These broader perspectives extend beyond constraints to include experiences of moral uncertainty, conflict and dilemmas, as well as emotional and ethical strain. This approach aligns with the wider literature, in which moral distress is increasingly framed as a psychological response to morally challenging events rather than solely to situations in which healthcare providers are unable to act in accordance with their values.^7 62^ However, research from Africa remains limited, particularly on moral distress-related concepts, such as moral injury.

#### Moral distress drivers and responses

That moral distress is a complex, multidimensional construct is reflected in the range of drivers highlighted in the reviewed papers that generate and shape moral distress. Individual-level factors such as perceived powerlessness, fear of infection and experience level clearly shape the experience of moral distress. However, the primary drivers of moral distress were at the organisational and systemic levels, including hierarchies, unclear directives, care rationing, supply shortages, high workloads, weak referral systems and limited managerial support, all of which are daily realities of care in many African public health systems.^28 37 51^ As highlighted in a nursing-focused review, moral distress arises from tensions between professional values and structural constraints, reflecting ethical strain within organisations, shaped by broader systemic inequities.^46^ Similar multilevel patterns are reported in high-income settings, where organisational factors (eg, hierarchy, support and regulations), interpersonal factors (eg, communication, teamwork and advocacy), resource constraints and rapid policy changes interact to shape how moral distress is experienced.^57 59^ However, in several African contexts, these drivers are influenced by chronic resource shortages and structural inequalities.

Although our search centred on moral distress and related concepts such as injury, we recognise similar experiences are described in the broader African literature, even where not explicitly labelled as moral distress. Resource constraints, relational dynamics (eg, leadership, norms and communication challenges) and cumulative trauma and unresolved dilemmas in fragile health systems have been shown to influence staff agency and psychological distress among healthcare providers.^58 63 64^ While these studies fall outside the scope of this review, they illustrate contextual conditions consistent with the organisational and system-level drivers identified in our findings.

Many papers in our review recommend interventions such as counselling, debriefing and ethics committees, as well as more structural reforms such as fair staffing and resource allocation (both often constrained by system-level financing and workforce policies).^28 37 46 49 51 54 56^ However, few papers document their implementation or evaluate the impact of these initiatives. More often described are healthcare workers’ informal strategies, like prayer and turning to family and peers to cope when formal support is absent.^46 51^ These informal mechanisms hint at healthcare providers’ efforts to build and maintain their resilience but also suggest a gap in the staffing levels and organisational preparation and support processes needed for safe reflection, peer support and shared learning about the ethical and emotional dimensions of care. Initiatives in Kenya, such as participatory leadership and emotional competence training, suggest that support processes can have a positive impact even in highly constrained environments,^65 66^ but these require sustained, multi-level efforts and should be implemented as part of broader change initiatives.

Literature from high-income settings increasingly recognises moral distress as a reflection of organisational ethical climate and workplace culture rather than of individual weakness.^67 68^ A growing body of evidence examines relational and organisational approaches to reduce moral distress and improve staff well-being and retention, such as Schwartz Rounds, structured ethics debriefings, facilitated discussions, education-based programmes and multidisciplinary ethics rounds.^20 21 69^ If and how such structured and systematic approaches might be implemented in more resource-constrained settings deserves further attention.

Globally, the importance of organisational strategies in protecting workforce well-being has been underscored in the COVID-19 pandemic literature. For example, Brown *et al*,^62^ through research conducted in Australia, identified the importance of personal (mindset, purpose, self-care), relational (teamwork, altruism, social support) and organisational (communication, leadership, policies, trust) sources of resilience among healthcare workers. These domains also resonate with African experiences beyond the pandemic and are discussed in a recent book chapter by Gilson and Barasa.^70^ As Gilson and Barasa note, ‘although individual agency, knowledge, skills and ability are important for resilience, the sum of such individual-level resources does not in itself represent organisational or system-level resilience’.

#### A multi-level framework for examining moral distress

Gilson and Barasa’s Everyday Health System Resilience (EHSR) theory^70^ provides a valuable lens for understanding the organisational processes underpinning resilience, complementing and extending Brown *et al*’s model.^71^ Drawing on organisational theory and empirical work in Africa, the framework examines how the personal, relational and organisational domains are shaped by ‘everyday’ system dynamics, not solely during acute health crises. It highlights the risks of relying on individual coping in contexts where organisational and structural conditions remain weak and offers insights into the opportunities and challenges of building relational and organisational resilience and how these interact with individual resilience and well-being. The framework also emphasises the importance of interactions between system ‘hardware’ (structures and resources) and ‘software’ (values, trust, power relations and leadership) in enabling resilience, and of three different types of organisational capacity: cognitive (collective sense making, shared purpose, problem-solving mindset), behavioural (collaboration, flexibility, learning) and contextual capacities (supportive networks, sufficient resources, distributed power).^70^

**Figure 2:**
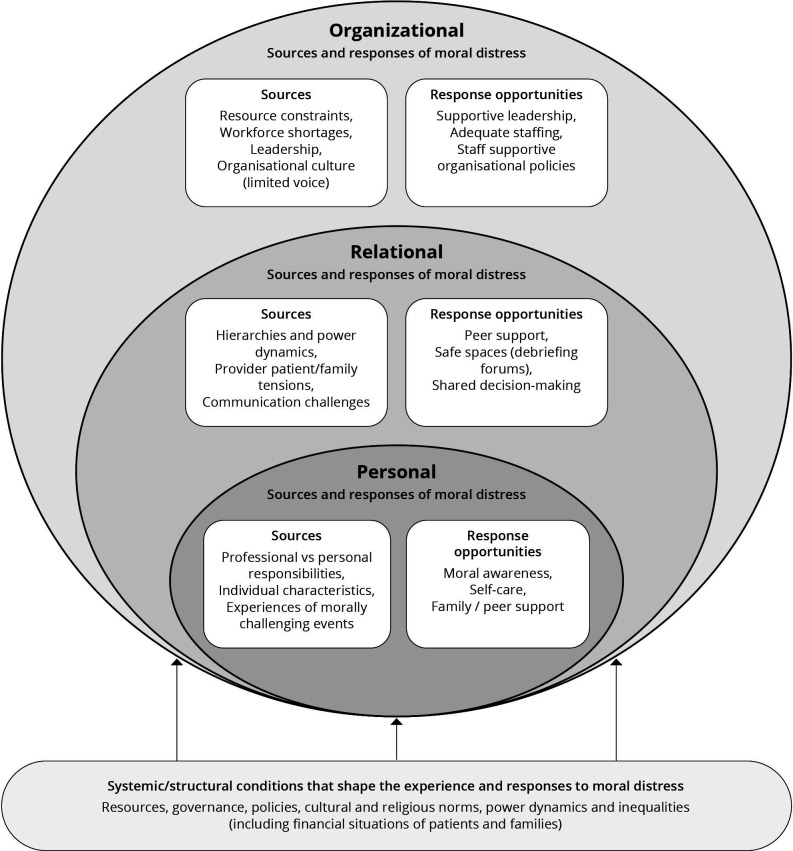
A multilevel framework for examining the sources of moral distress and opportunities for response/interventions.

Drawing on these insights and our review findings, we adapted Brown *et al*’s model^70^ to illustrate the multilevel influences shaping experiences of moral distress in African health systems (figure 2). Our conceptual framework comprises three nested domains (personal, relational and organisational), each encompassing sources of moral distress and opportunities for response, situated within wider systemic and structural conditions. At the personal level, sources of moral distress include conflicts between professional and personal responsibilities, individual characteristics (eg, age, gender) and experiences of morally challenging events, with opportunities for response such as moral awareness, self-care, peer and family support. At the relational level, sources include interprofessional relationships shaped by communication challenges and hierarchies, as well as tensions within healthcare teams and with patients and families, while opportunities for response include peer support, safe spaces for ethical reflection (eg, unit-level debriefing forums) and shared decision-making. At the organisational level, sources include resource constraints, workforce shortages, leadership and organisational culture (eg, limited staff voice), while opportunities for response include supportive leadership, adequate staffing and staff-supportive organisational policies. Consistent with Ramsey *et al*,^72^ the systemic and structural conditions include resource constraints, governance arrangements, power dynamics, cultural and religious norms and wider inequities that shape experiences across all levels.

While Brown *et al*’s framework focuses on personal, relational and organisational sources of resilience among healthcare workers,^71^ our adaptation reorients these domains to examine sources of moral distress and opportunities for response identified in this review, while also situating them within wider systemic and structural conditions emphasised within the EHSR framework.^70^ By bringing these two bodies of work together, our framework underscores how moral distress may arise from interactions across multiple levels of the health system and that there are opportunities for response across personal, relational, organisational and systemic levels through peer support, shared decision-making, supportive leadership and organisational policies that create space to recognise and address moral distress.

In summary, our framework helps illustrate how moral distress is produced and sustained in practice and highlights how organisational capacity for collective sense-making, as well as behavioural and contextual capacities, shape how morally distressing situations are collectively recognised and addressed at multiple levels. It underscores the need for multifaceted organisational and systemic responses that proactively address moral distress, prevent its escalation into clinical or organisational harm and, ideally, support wider organisational and system-level resilience.

### Strengths and limitations

This review offers a broad overview of how moral distress has been conceptualised, discussed and addressed in various African healthcare contexts. Although nursing papers predominated, consistent patterns in the organisational and systemic drivers of moral distress were identified across professions and healthcare settings. However, the search was limited to moral distress and related concepts and focused solely on English-language papers. This may have excluded relevant terminology, Francophone and Lusophone African papers, or studies in which moral distress was described indirectly or identified as a secondary rather than a primary finding. Additionally, screening was primarily conducted by a single reviewer, with only a 10% independent check, which could introduce selection bias and potentially omit relevant studies. Furthermore, most papers were cross-sectional and descriptive, providing limited evidence on the implementation and evaluation of the reported interventions.

### Implications for research and practice

The implications are both conceptual and practical. Conceptually, moral distress in African settings should not be viewed as an individual psychological burden but as a sign of ethical pressures stemming from organisational and systemic structures, consistent with findings from high-resource settings and potentially most pronounced in any context where health systems face persistent resource limitations and structural inequities. Exploring how moral distress arises and is managed, how organisational factors (such as leadership processes and practices) influence these experiences and whether and how health providers engage with support processes should support the development of organisational-level or system-level change processes that help minimise and manage moral distress. In practice, this could include strengthening opportunities for debriefing, peer support and shared problem-solving, as well as leadership and organisational processes that enable healthcare workers to discuss and address moral and ethical concerns. The latter may be limited by and require addressing broader system-level influences, such as staffing levels. In addition, future work should examine how informal coping mechanisms, such as prayer, peer support and family support, interact with cultural and community norms, and how these context-specific strengths can be integrated into organisational responses without being undermined by formalisation.

## Conclusion

Moral distress among frontline healthcare providers in African contexts is increasingly acknowledged in research on African health systems. The existing literature suggests it is inconsistently defined and inadequately managed within organisations. Our review and analysis highlight the importance of understanding moral distress as arising from the interaction of individual agency, professional relationships, organisational dynamics and broader systemic issues. This broad framing highlights the value of moral distress as a lens for recognising system-level stressors on individuals’ well-being and functioning, as well as system functioning, and for advocating for more ethically supportive work environments. Future research should prioritise understanding the organisational drivers of moral distress and the change processes that can reduce moral distress and strengthen health system resilience.

## Supplementary material

10.1136/bmjgh-2025-023358 online supplemental appendix 1

10.1136/bmjgh-2025-023358 online supplemental file 1

## Data Availability

All data relevant to the study are included in the article or uploaded as supplementary information.
