# Supplementary material for: Mapping the conceptualisation, contributing factors and interventions of moral distress among healthcare providers in Africa: a scoping review
Source: BMJ Glob Health. 2026 Aug 4;11(8):e023358. doi: 10.1136/bmjgh-2025-023358 (PMC13448609; doi:10.1136/bmjgh-2025-023358)
Supplement: Supplementary file 1 [file bmjgh-11-8-s001.docx]

**Appendices**

*Table A1: Eligibility criteria and Search terms*

| **PCC** | **Inclusion Criteria** | **Exclusion Criteria** | **Search terms** |
| --- | --- | --- | --- |
| **Participants** | Frontline health workers (e.g., nurses, doctors/physicians, pharmacists, social workers, allied health professionals) in hospital or community-based care. | Clinical research staff, patients, or family caregivers. | - (Health or healthcare or "health care" or hospital) adj (practitioner* or professional* or worker* or provider* or personnel*) OR - physician* or doctor* or clinician* OR - nurse* or nursing OR   ("Social worker*" or social) adj worker*) or pharmacist* or "allied health professional") |
| **Concept** | Studies focused on moral distress or closely related constructs (e.g., moral injury, constraint, uncertainty, dilemma), including those describing conceptual frameworks, drivers, and or interventions. | Studies that do not explicitly examine moral distress or its related concepts. | - moral distress OR moral* adj (dilemma* or challeng* or injur* or uncertaint* or conflict* or constraint*) |
| **Context** | Studies conducted in Africa (as defined by the World Bank classification), English language only. | Studies conducted outside Africa. Publications in other languages | - Africa* OR Kenya OR Uganda OR Tanzania OR Rwanda OR Burundi OR "South Sudan" OR Sudan OR Ethiopia OR Somalia OR Djibouti OR Eritrea OR Nigeria OR Ghana OR Senegal OR "Sierra Leone" OR Liberia OR "Côte d'Ivoire" OR "Ivory Coast" OR Togo OR Benin OR "Burkina Faso" OR Mali OR Niger OR Gambia OR Guinea OR "Guinea-Bissau" OR "Cape Verde" OR Chad OR Cameroon OR "Central African Republic" OR Gabon OR "Republic of the Congo" OR "Democratic Republic of the Congo" OR DRC OR Angola OR Zambia OR Zimbabwe OR Malawi OR Mozambique OR Madagascar OR Comor* OR Seychelles OR Mauritius OR Namibia OR Botswana OR "South Africa" OR Lesotho OR Eswatini OR Swaziland OR Morocco OR Algeria OR Tunisia OR Libya OR Egypt |

Table A2: Definitions of moral distress across included papers

| **ID** | **Author(s), Year** | **Title** | **Aims/Objectives** | **Moral Distress Definition (as stated/operationalised)** | **Conceptual framing** |
| --- | --- | --- | --- | --- | --- |
| 1 | Budden, 2024 | Audiologists' perceptions of ethical climate and level of moral distress in the provision of amplification services in South Africa | Explore HHPs level of moral distress and perceptions of workplace ethical climate | “Referred to psychological distress occurring from acknowledging moral responsibilities which are unable to be carried out as a result of perceived or actual constraints; thus, individuals may be forced to act in a way which contradicts personal values and ethical principles; a psychological response to challenging ethical events” | Constraint-based |
| 2 | Ashuntantang et al., 2022 | Bedside rationing and moral distress in nephrologists in sub-Saharan Africa | Overview of challenges in managing patients needing dialysis | “a specific psychological response to morally challenging situations such as those of moral constraint or moral conflict, or both” | Broad |
| 3 | Jaffre & Lange, 2021 | Being a midwife in West Africa: Between sensory experiences, moral standards, socio-technical violence and affective constraints | Examine midwives’ perspectives on profession, obstetric practices and personal lives | Not explicitly defined; explores moral standards/constraints | No explicit definition |
| 4 | Boakye et al., 2021 | An examination of the moral habitability of resource-constrained obstetrical settings | Examine moral habitability and influence on moral agency using Walker’s framework | Discusses moral agency/habitability; MD not explicitly defined | No explicit definition |
| 5 | Mwenya et al., 2024 | Determinants of moral distress among healthcare providers working in oncology department, Kenyatta National Hospital, Nairobi City County, Kenya | Describe determinants of moral distress among oncology providers | “Occurs when persons are aware of the correct moral actions but feel incapable of performing it. Also operationalised via MDS-R | Constraint-based |
| 6 | Ulrich, 2014 | Ebola is causing moral distress among African healthcare workers | Opinion piece on Ebola-related moral distress | “Result from not acting in accordance with what you believe to be ethically correct in professional practice”  “Occurs when doctors and nurses feel powerless to voice their concerns, do not agree with treatment procedures, or lack the equipment or resources necessary to advocate for patients. It can manifest as a sense of dread and a feeling of a loss of professional integrity” | Constraint-based |
| 7 | Solum et al., 2012 | Ethical problems in practice as experienced by Malawian student nurses | Explore students’ experiences of ethical problems during clinical practice | Focus on ethical problems; MD definition not explicitly stated, though it emerged in the findings | No explicit definition |
| 8 | Abdeen & Attia, 2020 | Ethical Work Climate, Moral Courage, Moral Distress and Organizational Citizenship Behavior among Nurses | Examine relationships among ethical climate, moral courage, moral distress, OCB | “An emotion that is explicated when the ethical complexity of a situation is not driving to solution” | Emotional dimension |
| 9 | Ohene et al., 2024 | Examining moral distress and injury resulting from the COVID-19 pandemic: Insights from the Ghanaian radiography workforce | Investigate level of moral distress and coping mechanisms among radiographers | “Emotional phenomenon that occurs when one knows the right thing to do for the patient, but institutional constraints make it impossible to pursue that action” | Constraint-based |
| 10 | Emmamally & Chiyangwa, 2020 | Exploring moral distress among critical care nurses at a private hospital in Kwa-Zulu Natal, South Africa | Determine frequency, intensity, overall severity of moral distress | “Refers to feelings of guilt, powerlessness and anger experienced by healthcare professionals in situations where they are unable to practice according to their professional and personal values” | Emotional dimension |
| 11 | Chukwuorji et al., 2024 | How Moral Distress Contributes to Depression Varies by Gender in a Sample of Sub-Saharan African Nurses | Examine link between moral distress and depressive symptoms; moderation by gender/age/experience | “The psychological disequilibrium and negative feeling experienced when a person makes a moral decision but does not follow through by performing it in overt behavior, or when one knows the right ethical decision to make but cannot make it due to institutional constraints” | Constraint-based |
| 12 | Kiddell-Monroe et al., 2018 | Humanitarian Ethics in MSF: Discussing Dilemmas and Mitigating Moral Distress | Discuss dilemmas and mitigating moral distress in humanitarian settings | “The manifestation of ‘traditional negative stress symptoms that occur due to situations that involve ethical dimensions and where the healthcare provider feels she/he is not able to preserve all interests and values at stake’” | Broad |
| 13 | Nagdee & de Andrade, 2023 | Moral injury in South African SLT&As due to patient death and dying | Explore moral injury experiences and coping among SLT&As in patient death & dying | Focus on moral injury; - “acting or witnessing acts that lead to moral transgression, which haslasting negative effects on one’s psycho-emotional, social, behavioural and spiritual well-being” | No explicit moral distress definition |
| 14 | Maluwa et al., 2012 | Moral distress in nursing practice in Malawi | Explore existence of moral distress among practicing nurses | “As pain or anguish from confronting a situation in which the person is  aware of a moral problem, acknowledges moral responsibility and makes a moral judgment about the correct action, but due to perceived constraints, acts in a manner that is perceived to be morally wrong.” | Constraint-based |
| 15 | Antwi & Galanza, 2024 | Moral caring competency and moral distress among Ghanaian nurses in adult care settings | Explore levels/relationship between moral caring competency and moral distress | Operationally defined as “a psychological discomfort that occurs when a nurse knows the correct course of action in morally challenging situations but is unable to act upon it.” | Constraint—based |
| 16 | Ntseke et al., 2023 | Moral distress among critical care nurses when executing do not resuscitate (DNR) orders in a public critical care unit in Gauteng | Explore and describe factors contributing to moral distress on execution of DNR orders | Context-specific accounts of what moral distress is; explicit definition not reported | No explicit definition |
| 17 | Addo et al., 2020 | Moral distress among midwives working in the labour ward of a hospital in Ghana | Explore and understand moral distress from midwives’ perspective | Occurs “when one knows the right thing to do, but institutional constraints make it nearly impossible to pursue the right course of action” | Constraint-based |
| 18 | Harrowing & Mill, 2010 | Moral distress among Ugandan nurses providing HIV care: a critical ethnography | Describe the manifestation and impact of moral distress among nurses providing HIV care | “The biopsychosocial, cognitive, and behavioural effects experienced by clinicians when their values are compromised by internal or external constraints it results from the inability to provide the desired care to patients” | Constraint-based |
| 19 | Getahun et al., 2024 | Moral distress and associated factors among nurses working in central Ethiopia: a cross-sectional study | Assess the magnitude of moral distress and associated factors | Operationalised via MDS-R; Nurses who were found to have greater than or equal eight symptoms from the 21 items moral distress scale revised (MDS-R) were considered as having moral distress and those who were found to have less than eight symptoms from the 21 items MDS-R were considered as not having moral distress | Constraint-based |
| 20 | Berhie et al., 2020 | Moral Distress and Its Associated Factors Among Nurses in Northwest Amhara Regional State Referral Hospitals, Northwest Ethiopia | Assess the proportion of moral distress and associated factors | “Is the cognitive-emotional dissonance that arises when one feels compelled to act against one’s moral requirements.” | Constraint-based |
| 21 | DeBoer et al., 2021 | Moral Distress and Resilience Associated with Cancer Care Priority Setting in a Resource-Limited Context | Describe moral experience and recommendations in oncology priority setting | “Arises when one is unable to act in accord with their ethical values because of external constraints, resulting in negative emotions” | Constraint-based |
| 22 | Langley et al., 2015 | Moral distress experienced by intensive care nurses | Explore and describe ICU end- of- life related moral distress | “A conflict which arises in certain circumstances to do with patient care, which occurs:   - when one knows or believes what the correct thing would be to do but can’t pursue this option OR - when either of two responses might be appropriate to a situation, both of which are not considered ideal” | Broad |
| 23 | Afoko et al., 2023 | Moral distress in nurses in developing economies: an integrative literature review | Review literature on ways nurses experience moral distress in clinical care settings | “a combination of (1) the experience of a moral event, (2) the experience of ‘psychological distress’ and (3) a direct causal relation between (1) and (2) which are necessary and sufficient conditions for moral distress” | Broad |
| 24 | Boakye, 2022 | No other alternative than to compromise: Experiences of midwives/nurses providing care in the context of scarce resources | In-depth analysis of resource scarcity influence on care and practice | Focus on moral agency, which is connected to the kinds of things for which people take responsibility. Therefore, moral distress is not defined. | No explicit moral distress definition |
| 25 | Beyaffers et al., 2020 | Predictors of moral distress among nurses working in Jimma University Medical Center, Southwest Ethiopia | Determine predictors of moral distress and relationships among variables | “The biopsychosocial, cognitive, and behavioural effects experienced by clinicians when their values are compromised by internal or external constraints; it results from the inability to provide the desired care to patients.” | Constraint-based |
| 26 | Dewey, 2016 | Providing care for many in the context of few resources: STS, burnout and moral distress in rural Uganda | Examine prevalence and sources of STS, burnout, moral distress | “The painful feelings and/or psychological disequilibrium that  occurs when one knows the right thing to do, but institutional constraints make it nearly impossible to pursue the right course of action” | Constraint-based |
| 27 | Sibiya & Rispel, 2025 | Unable to dodge the bullet: ethical dilemmas and moral distress of critical care nurses during COVID19 in a South African Province | Explore ethical dilemmas and moral distress of critical care nurses during COVID19 | “a stress that health professionals may experience when various obstacles prevent them from performing what they believe is ethically correct” | Constraint-based |
| 28 | Voget, 2017 | Professional nurses lived experiences of moral distress at a district hospital | Understand lived experiences of moral distress | Operationally defined as “Painful feelings and/or the psychological disequilibrium that occurs when nurses cannot carry out morally appropriate actions that a situation requires due to institutionalised obstacles” | Constraint-based |
| 29 | Afoko et al., 2022 | Moral distress experienced by neonatal intensive and paediatric care nurses in Northern Ghana: a qualitative study | Investigate how moral distress is experienced and supports offered by nurse managers | “‘when one knows the right thing to do, but institutional constraints make it nearly impossible to pursue the right course of action” | Broad |
| 30 | Good et al., 1999 | Clinical realities and moral dilemmas: Contrasting perspectives from academic medicine in Kenya, Tanzania and America | Discusses care for patients during the HIV/AIDS epidemic, and how academic medicine responds to overwhelming patient loads, resource scarcity, and political-economic constraints | Consistently focuses on moral dilemmas. Moral distress not explicitly discussed or defined | No explicit definition |

Table A3: Definition of moral distress related constructs

| Concept | Definition | Source Articles |
| --- | --- | --- |
| Moral injury | Harm to one’s conscience when a person perpetrates, witnesses, or fails to prevent acts contrary to their moral beliefs, values, or ethical codes; results in lasting psycho-emotional, social, behavioural, and spiritual harm. | Nagdee & Manuel de Andrade (2023); Ohene-Botwe, Antwi et al. (2024) |
| Moral standards | Shared ethical values and expectations that guide professional behaviour; in West African midwifery, these moral standards are entwined with gender norms, social hierarchies, and the moral meaning of childbirth. | Jaffré & Lange (2021) |
| Moral constraints | External or internal barriers, such as resource constraints, institutional hierarchies, or conflicting power relations, prevent health workers from acting in accordance with their moral judgment. | Boakye et al. (2022); Budden (2023); Ohene-Botwe et al. (2024) |
| Moral agency | The ability and freedom of individuals to make and enact moral choices consistent with their professional and ethical commitments, even in the face of adversity. Resource scarcity and workplace hierarchies often undermine this agency. | Boakye (2022); Boakye et al. (2021); Abdeen & Attia (2020) |
| Moral habitability | The degree to which a work environment enables moral agency and supports professionals' ethical identity. A morally uninhabitable environment is characterised by oppression, incoherent moral responsibilities, and distress. | Boakye et al. (2021) |
| Moral competence (or moral caring competency) | The integration of cognitive, affective, and behavioral abilities that enable nurses to recognise, deliberate, and act on moral issues in patient care. Low moral competence correlates with higher moral distress. | Antwi & Galanza (2024) |
| Moral courage | The capacity to uphold moral and ethical principles and act in accordance with one’s values even when doing so may involve personal or professional risk. | Abdeen & Attia (2020) |
| Ethical problems | Situations involving value conflicts, uncertainty, or moral tension where healthcare providers struggle to reconcile patient rights, professional duties, and contextual realities. | Solum, Maluwa & Severinsson (2012) |
| Humanitarian ethics | The reflective practice of navigating ethical dilemmas in humanitarian settings, creating spaces for open dialogue and support to reduce moral distress among aid workers. | Kiddell-Monroe et al. (2018) |

Table A4: Key drivers/contributors of moral distress

| Level | Types of Drivers | Representative Papers | Illustrative Quotes |
| --- | --- | --- | --- |
| Individual | Emotional burden, guilt, trauma | Maluwa et al. (2012); Harrowing & Mill (2010); Boakye et al. (2021); Dewey (2016) DeBoer et al. (2021) | *“it’s very stressful. You sort of play God when you’re choosing who gets to get treatment.” (DeBoer et al., 2021)* |
|  | Perceived incompetence, lack of training, limited confidence | Langley et al. (2015); Dewey (2016); Antwi & Galanza (2024); Emmamally & Chiyangwa (2020) | *“…inexperienced nurse, critically ill patient, not even getting support from senior staff.” (Langley et al., 2015)* |
|  | Fear for personal/family safety (outbreaks) | Ulrich (2014); Ohene et al. (2024) | *“Should clinicians protect themselves and their immediate family members from harm by walking away from their patients?” (Ulrich, 2014)* |
| Organizational | Resource scarcity, lack of supplies/equipment | Maluwa et al. (2012); Boakye et al. (2021); Ashuntantang et al. (2022); DeBoer et al. (2021); Boakye (2022) | *“I see that it affects us; it makes us ineffective; it makes nursing work difficult; it makes it frustrating at times, and then depressing or psychologically traumatizing because you see patients dying as a result of lack of basic consumables or items to actually work on the patient .” (Boakye et al., 2021)* |
|  | Staff shortages, heavy workload, high patient-to-staff ratios | Maluwa et al. (2012); Boakye (2022); Dewey (2016); Emmamally & Chiyangwa (2020) | *“...a woman who had a normal delivery . . . on discharge was supposed to be offered education on care of her new baby. I was alone on duty, and I was busy with other patients, this patient could not wait for me. She went home without any information . . . I felt I did not do anything . . . I felt very bad ...I had guilty conscience...(Maluwa, 2012)* |
|  | Unsupportive leadership, limited staff voice | Maluwa et al. (2012); Boakye et al. (2021) | *“When nurses raise these issues … they were labelled as trouble makers, hence preventing them from expressing themselves.” (Maluwa et al., 2012)* |
| Relational | Poor interprofessional communication, hierarchical workplace cultures, exclusion from decision-making | Boakye et al. (2021); Langley et al. (2015); Emmamally & Chiyangwa (2020); Maluwa et al. (2012); Addo et al. (2020) | *“Sometimes they [doctors] come to review without just asking anything. It kind of makes you feel like it is their work, they know it, and you have no idea about what they are doing. They are superior to you. Even if he asks you what at all are you going to tell him. He feels like he is knowledgeable in that aspect more than you are and there is no point in even asking you of your opinion. you have no idea.” (Boakye et al., 2021)* |
|  | Ambiguous or conflicting clinical directives, end-of-life decision-making, perceived treatment futility | Ntseke et al. (2023); Langley et al. (2015); DeBoer et al. (2021); Afoko et al. (2023) | *“Orders are not clear: one (doctor) will say “no escalation”, the other will say “give voluven” (a plasma volume replacement); there is no clear-cut end. You don’t know because there is no handover from one to the other” (Langley 2015)* |
| System | Poverty and inability to pay for care | Ashuntantang et al. (2022); DeBoer et al. (2021); Boakye et al. (2021) | *“What do we do with children with AKI who have no financial means, because we could save them?” (Ashuntantang et al., 2022)*  *“I want to raise the level of poverty, and we call it no money syndrome. They cannot actually buy the extra medications that are not covered by the health insurance or run the extra lab requested. So, you see that patient going down and you know that this patient could have survived with the appropriate antibiotics and the appropriate treatment to her, but you see that the patient will just go and then it would add to our mortality rate. This is a clear case of preventable maternal mortality.” (Boakye et al. 2021)* |
|  | Health-system financing limits, corruption, political interference | Ashuntantang et al. (2022); Dewey (2016) | *“The dialysis budget is badly used, Corruption ++ … Often politicians will interfere with our guidelines.” (Ashuntantang et al., 2022)* |
|  | Inequities | DeBoer et al. (2021); Ashuntantang et al. (2022); | *“We calculated how many lives we lose every year because we don’t have trastuzumab. Those are people who are dead who could be alive. But if you look at the cost of trastuzumab versus the main income in the country or spending per capita on health care, it’s off the charts.” (DeBoer et al., 2021)* |
|  | Cultural and family decision-making norms | Boakye et al. (2021); | *“Let me call my husband, my father-in-law to consult them first or they have to see a fetish priest before they agree. You feel so frustrated because the woman would not agree until the husband says yes. Wait for my husband to come.” (Boakye et al., 2021)* |
|  | Humanitarian and political access barriers | Kiddell-Monroe et al. (2018); Ulrich (2014) | *“We were blocked from reaching thousands of people … That’s morally distressing.” (MSF, cited in Kiddell-Monroe et al., 2018)* |

Table A5: Interventions to minimize and manage moral distress

| **Level of Intervention** | **Intervention / Support Type** | **Papers Reporting** | **Implementation status** | **Illustrative Quote (Source)** |
| --- | --- | --- | --- | --- |
| Individual | Counselling, stress management, debriefing | Ohene et al., 2024; Emmamally & Chiyangwa, 2020; Langley et al., 2015 | Limited implementation/Proposed | *“We are given the opportunity to maybe talk to a counsellor, but it doesn’t happen. Since the unit is always very busy, you suffer in silence…” (Langley et al., 2015)* |
|  | Peer and collegial support | Ashuntantang et al. (2022); Maluwa et al. (2012) | Participants reported implementation (informal) | *“Seventy-four percent of respondents did, however report having frequent discussions with colleagues about ethical dilemmas.” (Ashuntantang et al., 2022)* |
|  | Spiritual and faith-based coping | Boakye et al. (2021); Harrowing & Mill (2010) | Participants reported implementation (informal/personal) | *“You leave it to God … you just forget and pray over it and just have to tell God to strengthen me.” (Boakye et al., 2021)* |
|  | Personal resilience and reflective acceptance | Boakye et al. (2021); Dewey (2016) | Participants reported implementation (informal/personal) | *“Whether you are sad or not, you have to put yourself together … if you allow that moodiness, it will affect the next client.” (Boakye et al., 2021)* |
| Organisational | Unit-level ethical reflection, case discussions, debriefing forums | Ntseke et al. (2023); Langley et al. (2015); Boakye (2021) | Proposed/recommended | *“…need for unit-based ethical discussion platforms and debriefing sessions for supporting critical care nurses involved in executing DNR orders.” (Langley et al. 2015);* |
|  | Formal ethics committees, ethics education and training | Maluwa et al. (2012); Boakye (2021) | Proposed/recommended | *“…institutions should have ethics committees that would remind and resolve ethical issues … and update nurses’ ethical knowledge.” (Maluwa et al., 2012)* |
|  | Improved staffing, equipment and PPE | Ohene et al. (2024); DeBoer et al. (2021) | Proposed/recommended | *“The measures … include the provision of sufficient resources in the radiology department … improved staffing levels … and the provision of emotional and psychological support.” (Ohene et al., 2024)* |
|  | Supportive supervision, fair management practices | Maluwa et al. (2012) | Limited/inconsistent/Proposed | *“Nurses expected that their managers would ensure availability of resources for quality patient care … arrange regular staff meetings; send nurses for trainings and workshops; avoid favoritism.” (Maluwa et al., 2012)* |
| System | Strengthened governance, leadership, and health financing | Ashuntantang et al. (2022); DeBoer et al. (2021); | Proposed/recommended | *“The dialysis budget is badly used, Corruption ++ … Often politicians will interfere with our guidelines on provision of dialysis.” (Ashuntantang et al., 2022)* |
|  | Professional body advocacy, national oversight | Maluwa et al. (2012); Kiddell-Monroe et al. (2018) | Proposed/recommended | *“They should sometimes visit us so that they appreciate how we work with limited resources.” (Maluwa et al., 2012)* |
|  | Transparent triage and priority-setting frameworks | DeBoer et al. (2021) | Partially implemented | “*We have been very careful at dissecting out where we thought we could have the biggest impact and focusing our efforts and resources …” (DeBoer et al., 2021)* |
