# Supplementary material for: Mapping the conceptualisation, contributing factors and interventions of moral distress among healthcare providers in Africa: a scoping review
Source: BMJ Glob Health. 2026 Aug 4;11(8):e023358. doi: 10.1136/bmjgh-2025-023358 (PMC13448609; doi:10.1136/bmjgh-2025-023358)
Supplement: Supplementary file 2 [file bmjgh-11-8-s002.docx]

### BMJ Global Health Author Reflexivity Statement

Adapted from Morton, B., Vercueil, A., Masekela, R., Heinz, E., Reimer, L., Saleh, S., Kalinga, C., Seekles, M., Biccard, B., Chakaya, J., Abimbola, S., Obasi, A. and Oriyo, N. (2022), Consensus statement on measures to promote equitable authorship in the publication of research from international partnerships. Anaesthesia, 77: 264-276. <https://doi.org/10.1111/anae.15597>

| **Study conceptualisation** | |
| --- | --- |
| 1. How does this study address local research and policy priorities? | We examined evidence on moral distress among frontline healthcare workers in Africa to inform policies on organisational support and staff wellbeing. The review concentrated on African studies, emphasising how moral distress is understood in these settings, its main causes, and suggested interventions. Our findings underscore the importance of practice and policy reforms to enhance support at the team, organisational, and systemic levels, thereby reducing reliance on individual coping strategies. The review also identifies gaps for future research, particularly regarding how organisational conditions contribute to moral distress and how context-specific changes can be designed and assessed within limited health systems. |
| 2. How were local researchers involved in study design? | All authors contributed to the conception and design of the review. Researchers based at African institutions (JJ, DO, and JN) led the development of the research questions, analytical focus, and inclusion criteria to ensure contextual relevance, with significant input from the other authors. |
| **Research management** | |
| 3. How has funding been used to support the local research team(s)? | JJ, the lead author, is a PhD student fully supported by the project. Additionally, resources from the project were utilised to aid collaborative research processes, such as access to review software (Covidence) and qualitative data analysis software (NVivo). |
| **Data acquisition and analysis** | |
| 4. How are research staff who conducted data collection acknowledged? | All research staff involved in screening, full-text review, and data extraction are acknowledged through authorship. Roles in screening, verification, and data charting are described in the methods section. |
| 5. How have members of the research partnership been provided with access to study data? | All partners had access to the data extraction tables, shared via a secure platform. The review protocol is also publicly available through OSF – (https://osf.io/vr3uq). |
| 6. How were data used to develop analytical skills within the partnership? | The analysis process was collaborative, with all authors participating in the thematic synthesis through bi-weekly analysis discussions. This approach enabled sharing of skills in scoping review methods across the teams. |
| **Data interpretation** | |
| 7. How have research partners collaborated in interpreting study data? | Frequent meetings facilitated collaborative analysis, synthesis, and interpretation. |
| **Drafting and revising for intellectual content** | |
| 8. How were research partners supported to develop writing skills? | JJ guided the writing process through multiple revisions, with mentorship and substantial input from all authors throughout. This improved analytical clarity and precise reporting, supporting JJ's skill development and the shared intellectual contribution among all authors. |
| 9. How will research products be shared to address local needs? | Through publication and presentation at both local and international conferences. |
| **Authorship** | |
| 10. How is the leadership, contribution and ownership of this work by LMIC researchers recognised within the authorship? | All authors contributed significantly to conceptualisation, analysis, interpretation, and writing. Author roles are clearly specified in the contribution statement. |
| 11. How have early career researchers across the partnership been included within the authorship team? | Yes, for example, JJ, the lead author, is a PhD student under the mentorship of DO, JN, YZ, and SM. |
| 12. How has gender balance been addressed within the authorship? | Yes, the authorship includes women and a man across various early, middle, and senior career roles. |
| **Training** | |
| 13. How has the project contributed to training of LMIC researchers? | Yes, through hands-on involvement in scoping review methodology, synthesis, and writing. |
| **Infrastructure** | |
| 14. How has the project contributed to improvements in local infrastructure? | Open sharing of materials such as review protocol and contributed research resources. |
| **Governance** | |
| 15. What safeguarding procedures were used to protect local study participants and researchers? | This was a scoping review; therefore, safeguarding focused primarily on team well-being, supported by reasonable timelines and work arrangements. |
